# Development of a user-oriented completely open *in vitro* diagnostics system

**DOI:** 10.1155/S1463924696000016

**Published:** 1996

**Authors:** Robert Jakob, Dieter J. Vonderschmitt

**Affiliations:** 1 T ECAN AG R&D Feldbachstrasse 80 Hombrechtikon CH 8634 Switzerland; 2 Institute of Clinical Chemistry University Hospital of Zurich Rämistrasse 100 Zurich CH 8091 Switzerland


Journal of Automatic Chemistry, Vol. 18, No. 1 (January-February 1996), pp. 1-5

### Development of a user-oriented completely open *in vitro* diagnostics system

**Robert Jakob**
*TECAN AG, R&D, Feldbachstrasse 80, CH 8634 Hombrechtikon, Switzerland*

**and Dieter J. Vonderschmitt**

*Institute of Clinical Chemistry, University Hospital of Zurich, Rämistrasse 100, CH 8091 Zurich*

*A fully-automated open* in vitro *diagnostics analyser is described. It is based on a pipetting robot, robotic manipulator and modular components. The paper examines the way the system was developed and looks at future trends in* in vitro *automated systems.*

### Introduction

Since the first description of radioimmunoassays (RIAs) [1], and especially in the last two decades, a large number of immunoassays have been developed. There has been a move away from radioisotope labelling for three main reasons:

(1) Personal safety (although this is often exaggerated).
(2) Short half-life (and therefore shelf-life) of reagents.
(3) Unsuccessful attempts to automate RIA [2].

Nephelometric determinations of antigens have become more popular because of new techniques (laser nephelometry) and automation, but its sensitivity is limited and it is not applicable to haptens. Homogeneous assays (EMIT, CEDIA, FPIA) have also become popular, for they can be handled much like ordinary chemical determinations with corresponding automatic equipment. However, the methodology is not as sensitive as heterogeneous methods [3]. In addition, the tests are produced commercially and analysts often wait a long time until certain parameters are ready for commercial applications (for example FT4, TSH, Troponin). This makes the analyst dependent on the manufacturer and small volume tests are not commercially viable.

In the past, RIA tests could easily be handled in the users' laboratories by iodine labelling. Today ELISA (and to some extent TRFIA) are the only methods that can be custom-made with reasonable effort and sufficient precision. The success of the ELISA 'sandwich technique' is based on the fact that enzyme labelled antibodies against the second antibody are commercially available. ELISA is the current technique employed for small volume parameters on a commercial basis.

To deal with a large variety of different ELISA tests, however, a flexible instrument is necessary; an instrument that is open, programmable by the user and able to handle automatically a large number of different tests (with different recipients, microtitration plates, different wash solutions and reagents, different incubation times with or without shaking). An early example is the Zymate Microplate Management System [4]. The first systems were very bulky and required significantly more time for integration into the laboratory environment [5].

It can now cost tens of millions of dollars to develop [6] if the developer cannot spin-off from existing systems. This paper examines the development process taking the GENESIS RMP from Tecan as an example. In addition, future trends and needs in automated *in vitro* diagnostic analysis are discussed.

### Development tools

After the initial phase of market research comes a process of wish-driven thinking. Visions have next to be matched to reality by writing the specifications of the analyser to be developed There are many tools for this—for example quality houses, requirements deployment and scoring techniques [7], but in many cases good common sense is best. Internal and external communications have to be encouraged to get a maximum of input about the desirable and feasible characteristics of the analyser. For GENESIS RMP, an advisory board composed of experts in the field from different countries was consulted for each development step of the system, starting with the specifications and ending with the first serial production.

Once the specifications of the analyser have been defined, implementation is the hardest part. A development schedule clearly helps to prevent deadlocks. However, a common error in planning is assuming 'best case scenarios'. A realistic assessment of the time schedule is *vital.* Quicker or slower than expected development times should ideally be rare.

Frequent redesign meetings, involving all personnel developing the analyser are important for focusing everybody's thinking onto the project. This is necessary, since people tend to deviate from the fixed goal over time. A redesign meeting is not an exam, but a means to get everybody on board again, with the boat going in one common direction (see figure 1).

Potential equipment failures and their effects on the system and the surroundings (personnel and patient data) are analysed and preventative measures devised during the development phase.

The biggest surprise for most developers comes towards the end of the development process, when hardware and software have to be combined. Extended $\alpha$ (in-house) and $\beta$ (outside the plant) testing and debugging needs to be done; $\beta$ testing involves checking the combination of hardware and software in real-world conditions [9].

* *Correspondence to Dieter Vonderschmitt.*

0142-0453/96 $12.00 © 1996 Taylor & Francis Ltd.

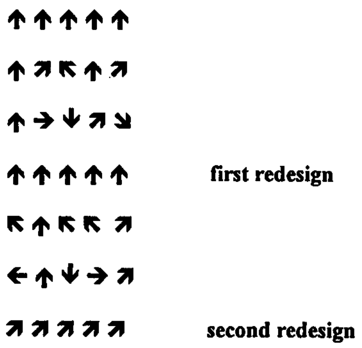

*Figure 1. The effect of frequent redesign meetings upon the orientation of the development team. Each arrow represents a member of the team. Over time, there is continuing risk of deviation from the common goal. Ideally, each redesign meeting helps to streamline the arrows. During a redesign meeting the common goal direction can change.*

### System development and system description

Scoring of the analyser specifications gave the highest ranking to the openness of the system; modularity was second. In fact, modularity should make the system more open, also servicing is easier.

The GENESIS RMP is a modular automated random-access analyser based on a pipetting robot as platform. A Cartesian robotic manipulator arm was chosen to allow objects to be transported in the system. Although this solution demanded a lot of space, it allowed objects like microtitration plates (MTPs), deep-well plates and reagent racks to be transported within the accessible work space of the robotic arm. The system could still be bench-top; its dimensions are 178 cm width, 77 cm depth and 87 cm height (see figure 3). A liquid dispenser with up to eight tips that can be equidistantly spaced between each other from 9 to 38 mm, supports any liquid handling movement from diverse sources to a variety of destinations (figure 2). To guarantee complete performance of ELISAs and agglutination assays, incubator/shaker, washer and reader (with four possible wavelengths, for example 405, 450, 492, 620 nm) are integrated components of the system. In order to keep the system open to the user, these can be exchanged.

Loading and discharging of reagents and consumables at the beginning and at the end of a run are the only manual interferences with the system, except for an eventual refilling of disposable tips (the system uses either standard Teflon®-coated steel-dispenser tips or disposable tips, or any combination of the two). The fact that the system needed to be bench-top size restricted the storing capacity of disposable tips on the analyser. This is a typical example of the kind of compromise necessary between two opposing development features of equal ranking.

In the case of standard tips, overflow-washing of the tips in a wash station can be programmed between each change of dispensed solution to avoid cross-contamination. The washing station comprises vertical columns with blind cavities in the middle. Washing is achieved by positioning the probe tip into the chamber and dispensing system liquid through it. This flushes the inside of the tip and the liquid flowing up the cavity and around the tip cleans its outside part. In the case of very adhesive solutions, a decontaminating intermediate washing step can be programmed. Whole microtitration plates, or plates partly filled with strips or individual wells, are loaded through an access-controlled loading port. For additional safety, the user has only limited access to the machine once a run has started (access is allowed upon request only and all moving parts of the system will stop). All this reduces user interference, which is a very significant cause of failures. Reducing user interference to the necessary minimum was considered essential.

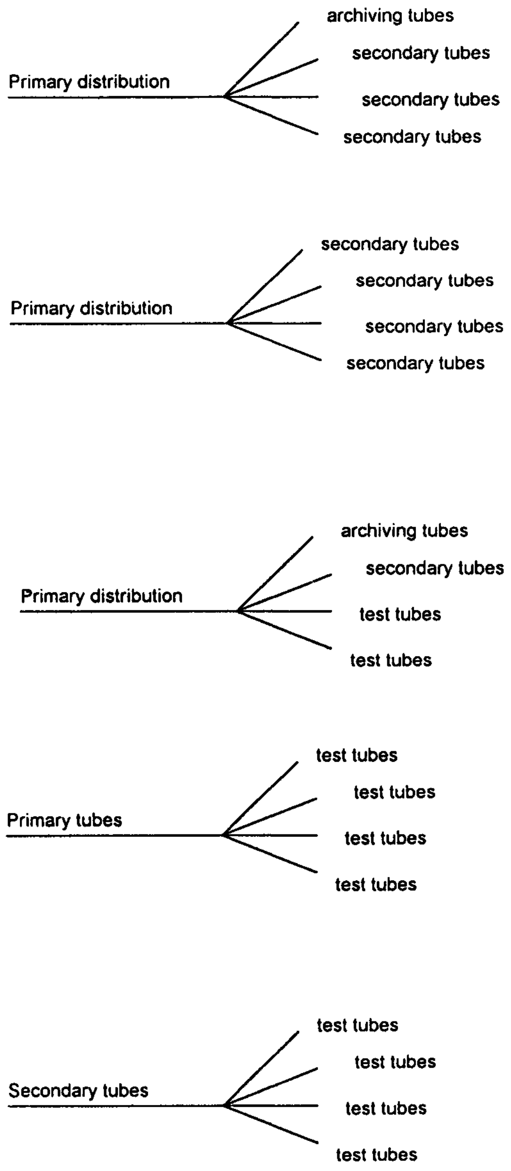

*Figure 2. The various pipetting strategies of the GENESIS RMP. The term 'tube' is used for any liquid compartment within the analyser worktable area. The system can work directly from (barcoded) primary tubes (10 to 17 mm). Within the same run it can pipet the solutions to be tested into test tubes whilst putting an aliquot aside for archiving (f.i. to build up a serotheque). The system can also be used to draw any combination of aliquots from primary tubes into secondary tubes (any size), which then could be used by different other analysers.*

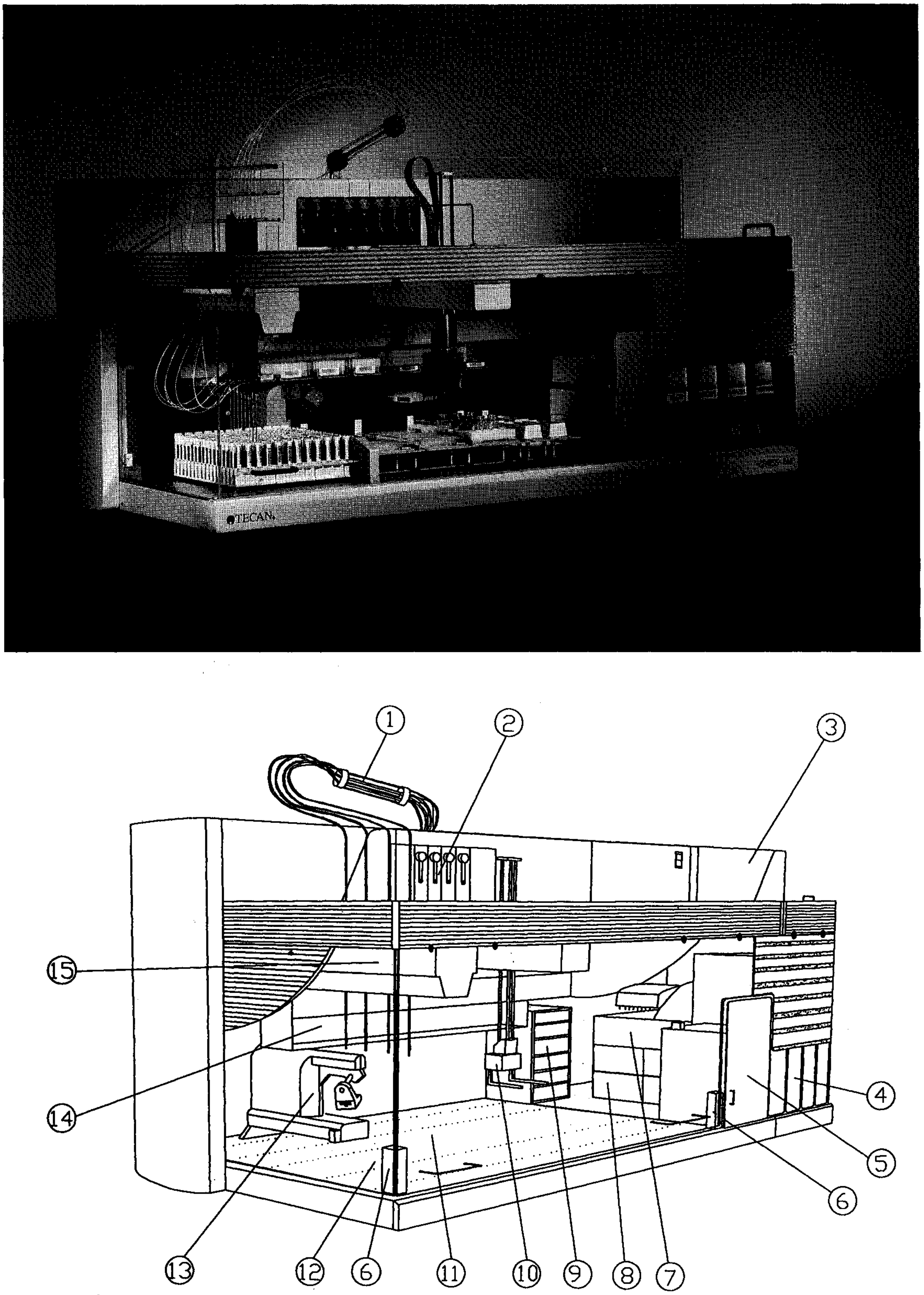

**Legend:**

1. Pipetting tubing
2. Precision pumps
3. Top safety panel
4. Wash liquid containers
5. Room temperature incubator
6. Access door lock
7. Washer
8. Reader
9. Incubator with optional shaker
10. Robotic Manipulator arm (RoMa)
11. Access door
12. Worktable
13. Positive identification (PosID)
14. Shelf
15. Liquid handling arm

*Figure 3. The GENESIS RMP (presented is the 150 model). The worktable containing primary tubes, wash station and various carriers for microtitration plates and reagents is empty in this diagram.*

The loading port (up to six plates can be processed at the same time) also serves as a darkened room temperature incubator, whereas the adjustable temperature incubator situated in the back of the machine (six individually heatable slots from room temperature +5°C up to 45°C) is also a linear shaker (1 to 8.2 Hz, 1 mm amplitude). This double function saves space and helped to reduce the analyser size to bench-top.

Exclusion of possible failure sources demands positive identification or better permanent identification [10] of samples and reagents. A laser bar-code scanner automatically reads the corresponding sample and reagent IDs and checks the correct worktable configuration. Consequently, the location of samples, processed samples, kits and global reagents are stored in a system data base log-file.

Additional safety is gained from automated clot-detection. The user can also opt for a photometric liquid dispense check if colour-coded reagents are used. This kind of automatic system check is important because of the ever growing legal consequences of product liability [11].

The user interface to the system is a Windows NT® based software that guides the user step by step through: set-up, service, method implementation, runs, data validation and evaluation, data export and data import. A log-file of all test specific data generated during a run is stored in the analyser's data-base, including eventual error messages and causes, again to comply with regulations. There is a scheduler in the software which determines the times necessary for the various dilution, pipetting, incubation, washing and reading steps for each test and then combines them to minimize overall run time. Up to six plates can be scheduled at the same time. Beta-site testing was done with a fixed scheduler (each panel of tests needs the same fixed maximal time span regardless of the number of samples processed). This fixed (simplified) scheduler should not influence the system performance, but it allowed $\beta$-testing to begin several months before the final software was released. As is often the case, the software development was behind schedule since the implementation of all the complex functions of the system demanded extensive programming resources.

### System integration

About 10 years ago, the first dedicated systems for automatic analysis with immunologic methods came onto the market. Today a considerable number of such systems with different test menus are commercially available (for example Tosoh AIA systems, ACS 180, Boehringer ES systems, Access, Axsym, Vidas, Autodelfia, COBAS CORE, Elecsys [12]). Test menus are offered according to the importance of bulk analysis and depending on the availability of reliable test methods. Test panels for thyroid function, fertility, tumour markers, serology and drugs of abuse are the most common panels, but no single instrument covers the whole programme. Tests with lower test frequency are executed manually or assisted by some kind of liquid sample handling systems.

Dedicated systems, as a rule, handle only one measuring protocol. An open system, however, is designed to be more flexible and to allow for a large number of different protocols to be executed (different reagents, different MTP formats, different washing solutions, incubation times, wavelengths etc.). The price for this is often a low-speed system compared to closed systems [13]. However, the user does not have to supervise the system and can do something else during this time.

In dedicated systems, reagents and hardware are often otpimized to exclude competitors' reagents and tests. In closed systems, reagents and consumables are matched to the machine. In this way, companies can pay off their investments in the development of an instrument through marketing and sale of reagents and consumables. Completely open systems, on the other hand, handle tests from different manufacturers and are freely programmable. A robotic manipulator acting in a Cartesian coordinate system has access to virtually every point of the usable workspace of the system or analyser. This allows for modular design according to the needs of the customer.

While dedicated systems, for economic reasons, are optimized to large volume parameters, open systems are not. In fact, it is easy to develop tests and run them on an open system.

In dedicated systems, the client is totally dependent on the manufactuer's strategy and ability to develop or supply new tests quickly. Some suppliers will not provide some parameters (for example serology) and in other cases only parts of a panel are realized (especially often within the thyroid function panel and the cardiac parameters). In the case of test supply problems (back order), the user has to look for quick alternatives. All this clearly limits the usefulness of closed systems for small laboratories.

Completely open systems are certainly more complex than dedicated systems, some of which resemble 'black boxes' with only a few buttons or keys to press and with very limited or no access to the mechanical and electronic parts. The complexity of open systems, however, is reduced through user-friendly software:

(1) The user is guided through the system and application by an intuitive software and graphical user-interface.
(2) Programming of test procedures has to be done just once during the system implementation and set-up procedure. During routine use operator interaction via display is generally reduced to pushing a few keys or a few mouse clicks.

Manual and semi-automated techniques can, therefore, be replaced by completely automated open systems that show the same comfort as dedicated systems. The detection system can be upgraded and could contain detectors working with fluorimetry, time-resolved fluorimetry and luminometry. Even the format of the reaction vessels is not fixed to the microtitration plate format, since the robotic manipulator can potentially handle a large variety of recipients.

### Future development

The pace at which immunologic tests develop depends partly on methods being available to produce monoclonal antibodies and available recombinant proteins. The number of both capture proteins is constantly increasing, opening the way to low volume testing of even the most special analytes. Although the MTP technique was originally thought to be for tests that yield either a positive or negative result, the current state of the art allows quantification. Not only has the number of proteins increased but so have the number of measuring techniques. ELISA tests with coloured products are widely used but more sensitive luminescent and fluorescent (for example TRFIA) markers are taking their share of the market. More recently, microtitration-plate based DNA analysis has been developed for clinical diagnostics [14].

The chief advantage of the system described in this paper is its flexibility. Conventional automatic equipment contains fixed programmes and fixed system layout and cannot be easily adapted to new techniques. A robotic equipment, however, is able to accommodate new and different peripheral devices. The robotic manipulator allows for a great variety of handling procedures within the analytical process and can thus be dedicated to new technologies and needs as they evolve, for instance when the classical and somewhat arbitrary [15] 96-well microtitration plate format will be replaced in the future by carriers of higher capacity and platforms for multiple analyte binding assays.

### References

1. Berson, S. A., Yalow, R. S., Bauman, A., Rothschild, M. A. and Newerly, K., *Journal of Clinical Investigation*, **35** (1956), 170.
2. Wheeler, M. J. In *The Immunoassay Kit Directory, Series A, Vol.* 2, *Part* 5 (Kluwer, Edinburgh, 1994).
3. Jakob, R., *Bioengineering*, **5** (1994), 16.
4. Hahn, G. D. and Lightbody, B. In *Advances in Laboratory Automation-Robotics*, Vol. 3, Strimaitis, J. R. and Hawk, G. L. (Eds), (Zymark Corp., Hopkinton, MA, 1987).
5. Hahn, G. D. In *Advances in Laboratory Automation—Robotics*, Vol. 7, Strimaitis, J. R. and Little, J. N. (Eds), (Zymark Corp., Hopkinton, MA, 1991).
6. Alt, P., *BMA Messenger*, **19**(2) (1992), 8.
7. Dean, B. V. and Nishry, M. J., *Operations Research*, **13** (1965), 550.
8. Wood, B. J. and Ermes, J. W., *Medical Device & Diagnostic Industry*, **1** (1993), 79.
9. Mowry, D. and Doll, G., *International Laboratory*, **9** (1994), 17.
10. Eggstein, M. In *Das Präsenzlabor* (Springer, Berlin, 1978).
11. Stürmer, U., Koepker, J. E. and Reischel, B. In *New EEC Product Liability—The U.S. in Comparison* (Gerling-Konzern, Cologne, 1988).
12. Naysmith, E. (Ed.), *The Immunoassay Kit Directory. Series A: Clinical Chemistry* (Kluwer, Edinburgh, 1994).
13. Jakob, R., *Hospitalis*, **1–2** (1993), 23–27.
14. Piiparinen, H. In *Laboratory Medicine* '95, 11th IFCC European Congress of Clinical Chemistry (Tampere, Finland, 1995).
15. Astle, T., *Screening Forum*, **3** (1995), 1.

